# A New Methodology for Defining Radon Priority Areas in Spain

**DOI:** 10.3390/ijerph18031352

**Published:** 2021-02-02

**Authors:** Alicia Fernández, Carlos Sainz, Santiago Celaya, Luis Quindós, Daniel Rábago, Ismael Fuente

**Affiliations:** Environmental Radioactivity Laboratory of the University of Cantabria (LaRUC), University of Cantabria, Santander, 39011 Cantabria, Spain; alicia.fernandezv@unican.es (A.F.); sainzc@unican.es (C.S.); luis.quindos@unican.es (L.Q.); daniel.rabago@unican.es (D.R.); fuentei@unican.es (I.F.)

**Keywords:** radon potential map, geography information systems, geology, risk

## Abstract

One of the requirements of EU-BSS (European Basic Safety Standards) is the design and implementation of a National Radon Action Plan in the member states. This should define, as accurately as possible, areas of risk for the presence of radon gas (^222^Rn) in homes and workplaces. The concept used by the Spanish Nuclear Safety Council (CSN), the body responsible for nuclear safety and radiation protection in Spain, to identify “radon priority areas” is that of radon potential. This paper establishes a different methodology from that used by the CSN, using the same study variables (indoor radon measurements, gamma radiation exposure data, and geological information) to prepare a radon potential map that improves the definition of the areas potentially exposed to radon in Spain. The main advantage of this methodology is that by using simple data processing the definition of these areas is improved. In addition, the application of this methodology can improve the delimitation of radon priority areas and can be applied within the cartographic system used by the European Commission-Joint Research Center (EC-JRC) in the representation of different environmental parameters.

## 1. Introduction

Numerous studies have shown that there is a clear correlation between indoor radon exposure and the risk of developing lung cancer [1,2]. Radon gas is considered to be the second leading cause of lung cancer after tobacco, and is responsible for between 3 and 14% of deaths caused by this disease in the first world [3,4] and the main source of ionizing radiation for the population [5,6,7,8].

The World Health Organization (WHO) recommended a reference level of 100 Bq/m^3^ annual average radon concentration to initiate action plans to minimize health hazards due to indoor radon exposure. However, if this level cannot be reached under the country-specific conditions, the chosen reference level should not exceed 300 Bq/m^3^, which represents approximately 10 mSv per year [4].

The interest in radon exposure maps is because the concentration of radon in buildings varies according to their geographical location. This variability is due to a large number of factors that affect the presence of radon indoors. These maps will be a useful instrument for applying the requirements of European legislation [5], which must be implemented in member states at all administrative levels: national, regional and local, to the radon problem. An overview of indoor radon mapping in Europe [9,10] showed the heterogeneity of the data: each country used different sampling strategies, measurement techniques, and representations of the data obtained.

In 2013, the EU-BSS (European Basic Safety Standards) required the design and implementation of National Radon Action Plans in the member states to identify areas of risk for the presence of radon gas in homes and workplaces. It establishes that the indoor radon concentration level in homes and workplaces be set at 300 Bq/m^3^, and requires the radon priority areas (RPA) to be delimited. The BSS defines the RPA as an area where it is expected that in a significant number of houses the average annual radon concentration exceeds the national reference level [5].

The problem was that different interpretations of RPA were introduced in each country [11,12,13,14,15,16,17]. In Europe, both mapping methodologies and the definitions of RPAs are diverse [18,19,20,21,22,23,24,25,26].

The concept of radon potential, soil radon potential, or geogenic radon potential is used by the different member states to define and delineate the Radon Priority Areas. Projects currently under development, such as the EURAMET MetroRADON [27,28,29], or the European Atlas of Natural Radiation [30,31,32,33,34,35], try to homogenize concepts, mapping methodologies that permit a clear definition of these areas.

The Spanish Nuclear Safety Council (CSN) published a radon potential map in 2017 [36,37]. The concept used in Spain, and defined by the CSN to identify “radon priority areas”, is that of radon potential. The CSN defines these areas using the 90th percentile to generate a cartography of the radon potential map of Spain. The variables used by CSN are the national ^222^Rn concentration database measurements in homes, geological information (lithostratigraphies), and exposure rates to terrestrial gamma radiation.

The CSN generated the radon potential map by combining these three variables: Radon measurements in homes were grouped by lithostratigraphic unit and level of exposure to gamma radiation, and units with homogeneous radon levels were obtained from these data groups. For these units, the 90th percentile (P90) of the radon concentration distribution was considered to be a limit with higher than 90% confidence, and the units were represented by rank in 5 categories based on radon levels from the use of the 90th percentile: P90 > 400 Bq/m^3^; P90 301–400 Bq/m^3^; P90 201–300 Bq/m^3^; P90 101–200 Bq/m^3^ and P90 < 100 Bq/m^3^. A radon concentration value is calculated using the 90th percentile, meaning that 90% of the values in an area are below that value, and 10% are above it. The CSN identified the areas of Spain where there are a significant percentage of homes with radon concentrations with a given probability of exceeding 300 Bq/m^3^ [36,37].

This paper sets out a mapping methodology that improves the definition of radon priority areas in Spain using the same variables used by the CSN (^222^Rn concentration measurements in homes, lithostratigraphies, and gamma radiation exposure data), but with a different approach. In addition, following the steps taken by the European Commission-Joint Research Centre (EC-JRC) in the production of the European Atlas of Natural Radiation and the European Radon Map [30,31,32,33,34,35], their reference coordinate system (the GISCO-LAEA projection) will be used and the 10 km × 10 km cell system to represent the data obtained. This cell system ensures the confidentiality of the radon samples taken in private homes and harmonizes the maps from the different countries.

## 2. Materials and Methods

### 2.1. Input Data

In order to produce a Spanish radon potential map with this new methodology, we used the following data sources:

#### 2.1.1. Concentration of ^222^Rn in Homes

There were 11,500 data points on radon concentration measurements throughout Spain used in the preparation of this study. These measurements are taken from the national ^222^Rn concentration database in homes carried out in sampling campaigns between 1991 and 2016 grouped by municipalities [38]. The samples were taken inside houses, on the ground floor, and the measurements were made with track detectors (CR39) exposed for a period of three to six months.

The bulk of these 9500 measurements were collected by the University of Cantabria through different projects sponsored by CSN according to the internal location protocol of the Environmental Radioactivity Laboratory of the University of Cantabria (LaRUC), created using the indications of the CSN Safety Guide 11.01 [39]. The LaRUC Laboratory has been validated by Public Health England (PHE) since 2002 [40] and accredited since 2016 through UNE-EN ISO/IEC 17025, ENAC [41], to carry out this type of radon measurement on air. This will be the dependent variable (variable 0) in this study.

#### 2.1.2. Gamma Radiation Exposure Data

The gamma radiation exposure data were obtained from the Natural Gamma Radiation Map (MARNA) [42]. This map assesses the rate of exposure to terrestrial gamma radiation at a height of 1 meter above the ground. It was produced by taking aerial and terrestrial measurements with a variety of analysis techniques, and these were later correlated through the MARNA project [43].

Terrestrial gamma radiation rates in Spain range from 44 to 287 nGy/h. This information is identified in 22 individualized rates. The information about the 22 terrestrial gamma radiation rates (rates of 44 at 287 nGy/h) in Spain was extracted after downloading the map image in high quality (.tiff) offered by the CSN website [42]. This will be the first independent variable (variable 1) analyzed.

#### 2.1.3. Lithostratigraphies

The Lithostratigraphic, Permeability and Hydrogeological Map of Spain at a scale of 1:200,000 [44] produced by the Geological and Mining Institute of Spain (IGME) was used, and 329 lithostratigraphic units were analyzed. This map includes the permeability of the lithological units, homogeneously representing the lithostratigraphies and grouping them by similar permeability values. This cartography is used because numerous studies show the importance of soil permeability in determining the radon potential inside buildings [45,46]. The digital cartography was downloaded in a compatible format (.shp) with the use of Geographic Information System (GIS) programs. This will be the second independent variable (variable 2) analyzed.

#### 2.1.4. Radon Potential

Information about radon potential in Spain was obtained after downloading the map image from the CSN website [11,36]. The 5 units shown were analyzed with homogeneous radon levels based on radon levels from the use of the 90th percentile: Unit 1 (>400 Bq/m^3^), Unit 2 (301–400 Bq/m^3^), Unit 3 (201–300 Bq/m^3^), Unit 4 (101–200 Bq/m^3^), and Unit 5 (<100 Bq/m^3^). This will be the variable (variable 3) used to perform the comparison of the data obtained in this work.

### 2.2. General Procedure

#### 2.2.1. Framework

A Geographical Information System program (ESRI ArcGis v. 10.0, Environmental Systems Research Institute: Redlands, CA, USA) [47] was used to produce the cartography for this paper. The KaleidaGraph v. 4.1 (Synergy Software: PA, USA) [48] program was used to analyze the data obtained and to make graphs.

To follow a similar scheme to other EU member countries, we began working with a continental level projection system (GISCO-LAEA), and defined the European working area with a 10 km × 10 km grid with established limits (coordinates) as suggested by the Joint Research Centre of the European Commission EC-JRC [30,33]. To define the Spanish working area, we used the administrative boundaries provided by the National Geographic Institute [49], generating a total of 5478 cells of 10 km × 10 km. For each cell, an identifying code was created and its centroid in meters (“x” and “y” coordinates) was calculated.

#### 2.2.2. Harmonization of Input Data

The formats in which the source information for these variables appears are different, and so it was necessary to harmonize this information to later process the data:

##### Concentration of ^222^Rn in Homes

The radon concentration variable was analyzed using the 11,500 data points obtained in the various measurement campaigns mentioned above. This information was stored in the GIS database and transferred to the 10 km × 10 km cell system: the transposition of the values into the cell system was performed by calculating the arithmetic mean of the radon concentration points data (in Bq/m^3^) contained in each 10 km × 10 km cell. This variable therefore contains information on 5478 fields in its attribute table, corresponding to the average radon concentration (in Bq/m^3^) for each of the 5478 Spanish cells. The decision to use the arithmetic mean was taken because the EC-JRC suggested it in the European Radon Atlas [30,31,32,33,34,35] as the most appropriate parameter in the representation of this variable, due to the great variability of the concentrations obtained per 10 km × 10 km cell and because it is used in most epidemiological studies.

##### Exposure Rate to Terrestrial Gamma Radiation, Lithostratigraphy and Radon Potential

The information about the Spanish lithostratigraphic units was downloaded in a shape format, and so we worked with geological data of the 329 polygons and the attribute table of lithostratigraphic units provided by IGME. The data for the rates of exposure to terrestrial gamma radiation and radon potential in Spain were downloaded in a high-quality image format. These images were georeferenced to the Spanish administrative boundaries, and its polygons were later digitized in as much detail as possible (at an approximate scale of between 1:3000 and 1:5000): the 5 units with homogeneous radon level were digitized as 5 polygons, assigning them their value in Bq/m^3^. There were 22 terrestrial gamma radiation rates digitized as 22 polygons, assigning them their value in nGy/h. A noteworthy fact is that there are no data on exposure to terrestrial gamma radiation for the Balearic Islands or the Canary Islands, so this variable could not be taken into account when conducting the study in these areas.

As mentioned above, each of these variables in shape format contains the graphical unit/polygon (field) information stored in its attribute table, with each field being a record with information about the typology of the element or surface coverage to be analyzed (it is a homogeneous category of information). For the subsequent data analysis, it was necessary to calculate, for each variable, which was the unit or field (polygon) with the largest surface area contained in each 10 km × 10 km cell. To do this, the cell system was intersected with the variables gamma radiation, lithostratigraphies and radon potential, and the surface of the majority field was calculated in each one. In this way, each 10 km × 10 km cell was assigned the value of the field with the highest probability of occurrence in those 100 km^2^.

#### 2.2.3. Data Processing

The data processing was different for the input data depending on the origin of the source information: for the dependent variable (concentration of ^222^Rn in homes) the arithmetic mean data of the radon concentration points (in Bq/m^3^) contained in each 10 km × 10 km cell was transferred to the cell system. The data for the independent variables (exposure rate to terrestrial gamma radiation and lithostratigraphy), and the data for performing the comparison of the data and validating the study results (CSN radon potential) were transferred to the cell system by the generation of density maps.

**Figure 1:**
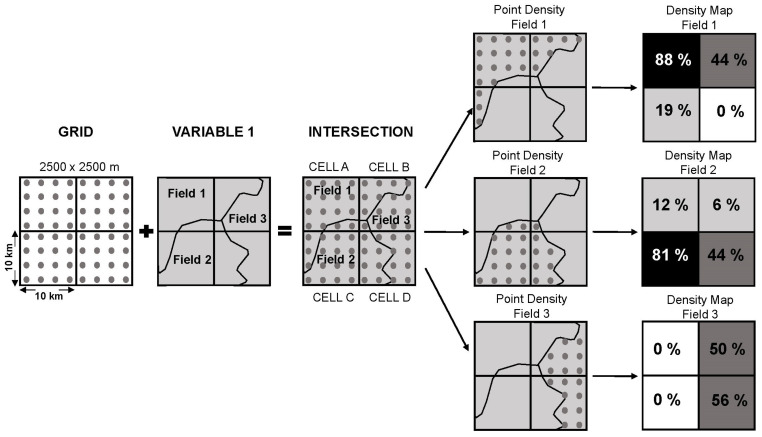
Diagram of the methodology for creating the density maps.

The methodology of the density map creation process is shown in Figure 1 and is as follows:

The first step was to create a 2500 m × 2500 m dot mesh on each side, fitted to the limits of the 10 km × 10 km cells in Spain. This meant that each cell was covered homogeneously by a total of 16 points. This dot mesh allowed the extraction, for each 10 km × 10 km cell, of the points contained in each field of the study variables. The process was carried out by intersecting the dot mesh with each of the previously selected fields; in this way a series of layers were created that indicated the density of points per 10 km × 10 km cell: the minimum value (0) corresponded to the absence of that field in that cell, and the maximum value (16) was related to the total presence of that field in that cell. Thus, 22-point layers related to variable 1 (exposure rate to terrestrial gamma radiation), 329-point layers were associated with variable 2 (lithostratigraphies), and 5-point layers were created for variable 3 (CSN radon potential). Density maps were generated with each of these point layers, and fitted the limits of the 10 km × 10 km cells from the point density tool [50].

Once the density maps of each variable were generated, the relationship between the dependent variable (concentration of ^222^Rn in homes) and the independent variables (exposure rate to terrestrial gamma radiation and lithostratigraphy) was analyzed. In previous steps, the centroid (“x” and “y” coordinates) of the 5478 cells 10 km × 10 km had been calculated, and an identification code added to each of them. Using the extract-by-points tool, the radon concentration value transferred to the cell system (dependent variable) was extracted. In the same way, with this tool, the value of the gamma rate information was extracted for each of the cells of the 22 density maps related to the fields of variable 1. The same procedure was carried out with the 329 fields of variable 2, extracting the information from the lithostratigraphic typology.

Using the data extracted, a simple linear correlation analysis was performed to check the positive or negative relationship between the two parameters for each cell. For example, in a cell with code 1 the Pearson correlation coefficient (R) of the average radon concentration is calculated, and so is the exposure rate to terrestrial gamma radiation with the field 44 nGy/h, for that same cell, the field 88 nGy/h and so on for each field of each variable. The degree of adjustment was quantified through the Pearson correlation coefficient (R), giving for each of the correlations, a value between −1 and +1. The data of these correlations were normalized into 9 categories: value 1 R > +0.75, value 2 (R +0.74 to +0.5), value 3 (R +0.49 to +0.25), value 4 (R +0.24 to +0.1), value 5 (R = 0), value 6 (R −0.1 to −0.24), value 7 (R −0.25 to −0.49), value 8 (R −0.50 to −0.74), and value 9 (R < −0.75). This grouping into ranges of values facilitated the process of representing the correlations obtained between the radon concentration with respect to exposure to gamma radiation and lithostratigraphy.

### 2.3. Development of the Relationship Maps between Independent Variables and the ^222^Rn Concentration in Homes and the New Radon Potential Map

For the variable terrestrial gamma radiation exposure rate, the data were represented graphically using the cell system, which gathers the 22 fields into 5 categories, defined by their radon concentrations: 44 nGy/h correspond to 100 Bq/m^3^ and 89 nGy/h with 300 Bq/m^3^ [51]. The correlations obtained between the radon concentrations with respect to exposure to gamma radiation were represented graphically in 9 categories according to the ranges mentioned above. Similarly, the lithostratigraphy variable was represented graphically in the cell system, bringing together the 329 lithostratigraphic fields of the Iberian Peninsula, the Balearic Islands, and the Canary Islands. The correlations obtained between the radon concentrations with respect to the lithostratigraphies were also represented graphically in 9 categories.

From these two correlation maps, a new radon potential map (Radon Potential Map Calculated) was generated. The sum of the categories of both maps was represented on this calculated map, and so the numerical range of each cell was between 1 and 18: Values from 13 to 18 indicate a positive linear relationship with radon and therefore a high probability of finding high concentrations. Values from 8 to 12 indicate the absence of a relationship and therefore an average probability of finding high concentrations. Values from 1 to 7 indicate a negative linear relationship with radon and therefore a low probability of finding high concentrations.

To facilitate the interpretation of the results, and to represent the data according to their possible radon concentration range, the values were reclassified into 5 categories: Category 1 (>400 Bq/m^3^), Category 2 (301–400 Bq/m^3^), Category 3 (201–300 Bq/m^3^), Category 4 (101–200 Bq/m^3^), and Category 5 (<100 Bq/m^3^). The equivalences applied to the entire process are shown in Table 1 below:

The methodology used to evaluate the results was as follows: the success or failure capacity per 10 km × 10 km cell was compared for each of the variables analyzed (^222^Rn concentration measurements in homes, exposure rate to terrestrial gamma radiation and lithostratigraphies), from both the CSN P90 Radon Potential Map and the Radon Potential Calculated Map. It was considered a success if a cell was in the same concentration or range of values (see Table 1 equivalences) and it was considered a failure if the cell was not a match.

## 3. Results

### 3.1. Analysis of Variables

#### 3.1.1. Concentration of ^222^Rn in Homes

Regarding the concentration of ^222^Rn in homes, the 11,500 data points on the concentration of ^222^Rn in air were analyzed using as a starting point the central trend measures and dispersion, and according to the shape of the distribution sample. Table 2 offers the updated data regarding previous publications [52,53] showing the main parameters obtained:

The central trend measures show that the data do not follow a normal distribution, since the arithmetic mean (101 Bq/m^3^) is far from the median (56 Bq/m^3^). Furthermore, the standard deviation of the arithmetic mean is 260.6 Bq/m^3^, which shows a high dispersion of the data.

**Figure 2:**
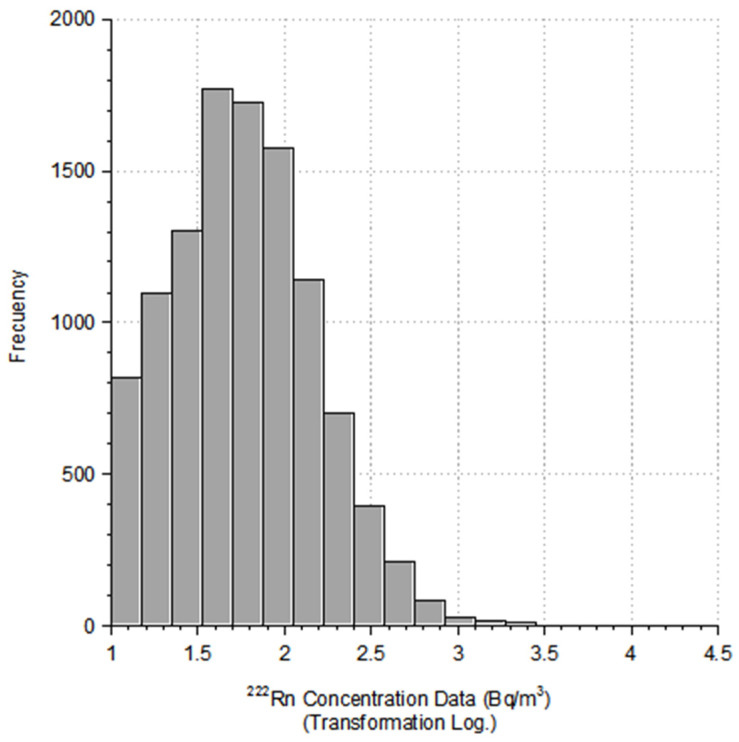
Histogram of the radon concentration data.

Analysis of the shape of the sample distribution shows a high coefficient of kutorsis (K = 1497), indicating a leptokurtic distribution, whereas the asymmetry coefficient (CS = 31.5) indicates a positive asymmetry: The distribution of measurements is log-normal. The distribution of measurements is log-normal, as shown in the histogram (Figure 2). This distribution is usual for radon concentration measurements since most of the measurements obtained are in low concentrations, whereas only a few measurements appear in the high concentration range.

Analyzing these 11,500 data points, it is seen that 27% of the samples exceed the level of 100 Bq/m^3^ to initiate action plans (5% of the measurements exceed 300 Bq/m^3^ and 22% are in the range of between 100 and 300 Bq/m^3^).

On transposing these data to the cell system, it is seen (Table 3) that the majority (76%) are in the range of low concentrations (<100 Bq/m^3^), 22% between 100–300 Bq/m^3^, and that the percentage of cells in high concentrations (>300 Bq/m^3^) is reduced to 2%.

It is also clear that the sampling in Spain is not heterogeneous, since there are areas where the measurement density is much higher than others; this is because the sampling in Spain was defined based on a series of criteria that concentrated the number of measurements in areas with potentially high radon concentrations. The decision of how many measurements to be carried out in each 10 km × 10 km cell was made by the CSN, taking into account the general objectives established by the EC-JRC in the creation of the European Radon Map, considering superficial, population and lithostratigraphic criteria, and according to the rate of exposure to terrestrial gamma radiation [51,52].

Despite efforts to try to cover the entire country with at least one measurement per 10 km × 10 km cell, it can be seen that a large part of its surface does not have any measurements (40%). Of the cells for which measurements are available, it is representative that a large percentage of Spain is covered with only one measurement (47%) or with two measurements (19%), whereas cells with more than 6 measurements represent 15% of the total.

Analyzing cell percentage according to concentration category and measurements, it is seen that 68% of cells with concentrations greater than 400 Bq/m^3^ have more than two measurements (in 52% of cases from 2 to 6 measurements, and in 16% more than 6 measurements), and that in the concentration range between 301 and 400 Bq/m^3^ this percentage of cells is also high (66%). The percentage is slightly lower for the intermediate concentrations (101–300 Bq/m^3^) where 63% of cells have more than 2 measurements. Low concentrations (<100 Bq/m^3^), despite being the most numerous category, is the one with the fewest measurements. Half of its cells have a single measurement, reducing the number of cells with more than 6 measurements to 9%.

It is clear that as the number of measurements per cell increases, the concentration ranges are better defined.

#### 3.1.2. Exposure Rate to Terrestrial Gamma Radiation

**Figure 3:**
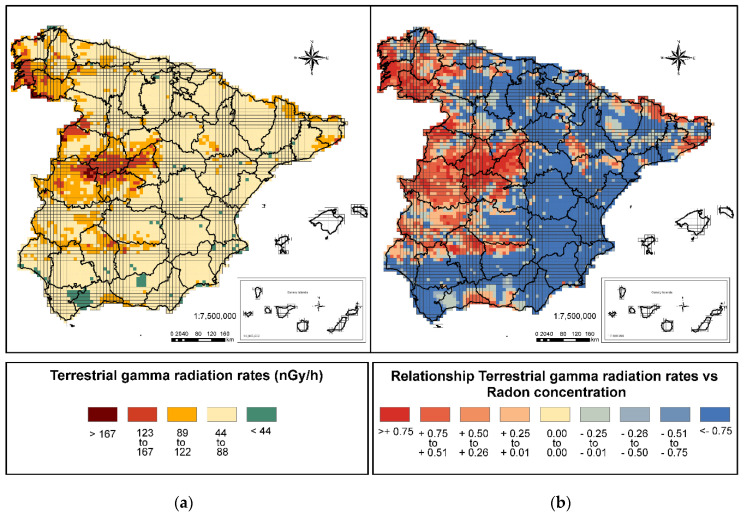
(**a**) Figure Terrestrial gamma radiation rates (nGy/h) and (**b**) Relationship between terrestrial gamma radiation rates and radon concentration.

From the analysis of the rates of exposure to terrestrial gamma radiation (Figure 3a), 95% of the peninsula is found to have medium and low rates: 2% of the country is below 44 nGy/h, and 93% between 45 and 122 nGy/h. The areas with rates higher than 122 nGy/h are few (5%), and are mainly in the northwest area of the peninsula and in the Central System. In these areas, there is a clear correspondence between the presence of high radon concentrations and high rates of gamma exposure [43,54].

Analyzing the data of this variable with respect to radon concentrations (Figure 3b), a positive linear relationship is observed between the two parameters starting at 78 nGy/h, the relationship becoming clearer in the case of the identified areas of high rates. These areas correspond once again to those previously mentioned, along with areas of the Catalan Coastal Cordillera and the Pyrenees, corroborating the correspondence of high radon concentrations and high rates of gamma exposure.

#### 3.1.3. Lithostratigraphies

Regarding the lithostratigraphy variable, it is known that the main indicator in determining a higher or lower probability of high concentrations of radon in an area is the presence of uranium in soils and rocks, for which reason the lithological formations with a high proportion of uranium will generate a high proportion of radium and therefore a higher proportion of radon. In general, the highest uranium values (>2.88 ppm) [55], are associated with acidic intrusive plutonic rocks such as granites.

**Figure 4:**
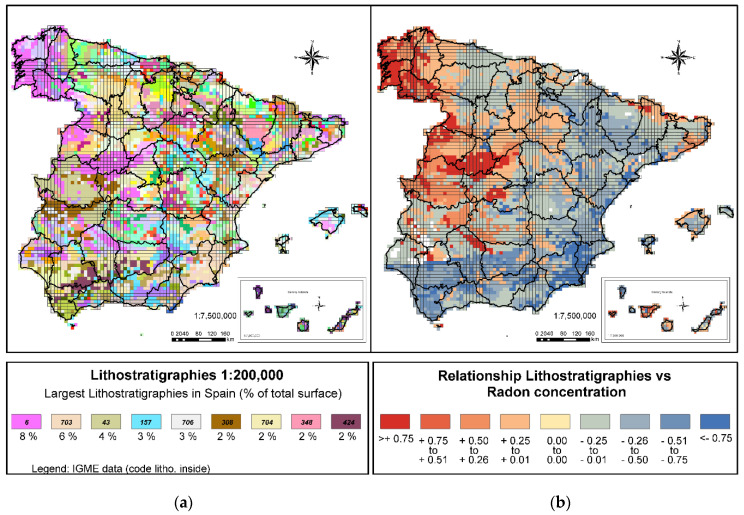
(**a**) Figure Lithostratigraphies 1:200,000 and (**b**) Figure Relationship lithostratigraphies and radon concentration.

The analysis of lithostratigraphies (Figure 4a) in Spain suggests that the geologies most commonly found in Spain are acidic plutonic rocks such as granites, granodiorites, and quartz diorites (8% of the territory). Due to the large number of lithostratigraphies present in Spain, the legend of this figure only shows the most numerous (more than 2% of the territory), the complete legend is available in the IGME [56]. It is also noteworthy that 4% of the territory is made up of slates and greywacke. Both shales (metamorphic rocks produced by silt-clay sedimentary rocks) and greywacke (detrital sedimentary rocks derived from the dismantling of acidic plutonic rocks) generally also have high uranium content [43].

In the areas where these two formations are present, there may be a high probability of finding high radon concentrations, which was confirmed after performing the correlation analyses of the two variables.

The results of the relation between lithostratigraphies and radon concentrations (Figure 4b) show that 100 lithostratigraphies show a positive relationship. The clearest relation (>+0.75) appears in a single case, in the geologies corresponding to acidic, Hercynian plutonic rocks (granites, granodiorites, quartz diorites). It has been confirmed that this geology is associated with a high presence of radon.

In addition, six other lithostratigraphies show a marked relationship with the presence of high radon concentrations (between +0.51 and +0.75), representing another 8% of the peninsular surface. They mainly correspond to metamorphic rocks such as shales, gneiss, schists, or quartzites (these are rocks with high concentrations of uranium) [43] and detrital sedimentary rocks such as greywacke derived from acidic plutonic rocks.

On analyzing the geographical distribution of these areas, it is seen that they correspond to the northwest area of the peninsula and the area of the Central System. A close relationship is also observed with the geological formations in the west of the peninsula and their extension towards Sierra Morena, specific areas of the Pyrenean Range, and in the area of the Catalan Coastal Cordilleras.

### 3.2. Comparison of Radon Potential Maps Generated

**Figure 5:**
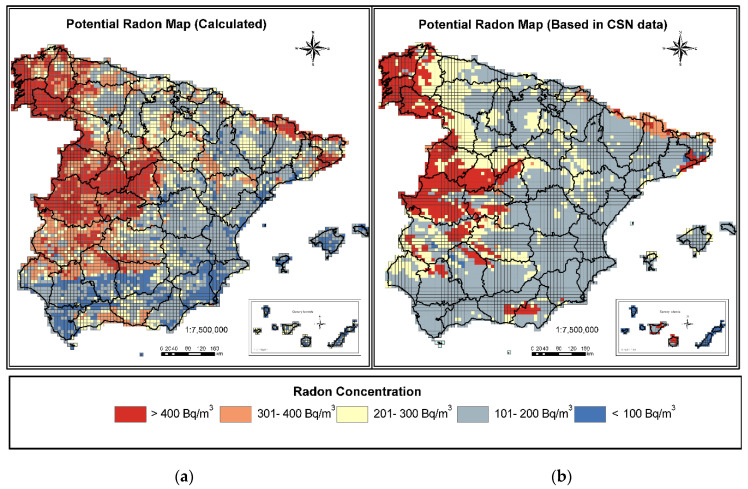
Potential Radon Map. (**a**) Calculated Map and (**b**) Map based on Spanish Nuclear Safety Council (CSN) data.

As shown in Figure 5, where the P90 Radon Potential Map generated by the CSN (hereafter P90 Potential Map) and the Radon Potential Map Calculated in the present study are compared, both maps show a similar percentage of cells in the range of radon concentrations greater than 400 Bq/m^3^ (17% in the case of the Calculated Map and 16% in the P90 Potential Map). In both maps, the areas defined in this range correspond once again to the northwest of the peninsula, the Central System area, the west of the peninsula extending towards Sierra Morena, south of the Pyrenees, and in the area of the Catalan Coastal Cordilleras.

The increase in the weight of the cells with concentrations between 301 Bq/m^3^ and 400 Bq/m^3^ is significant: it goes from 2% in the P90 Potential Map to 19% in the case of the Calculated Map. As will be seen later, the calculation of this new zoning fits possible radon concentrations more reliably. It is mainly seen in the west of the peninsula surrounding the highest concentrations. The area of Sierra Morena up to the border with Portugal is also clearly defined in this range, and in some areas to the north of the peninsula, areas of the Penibaetic System or areas to the west of the Ebro valley.

Both maps have a similar percentage of cells in the concentration range between 201–300 Bq/m^3^ (20% in the case of the Calculated Map and 21% in the P90 Potential Map). However, analyzing Figure 4 shows changes in zoning: in the CSN P90 Potential Map these areas were defined as mainly bordering the areas of greater concentrations in the west of the peninsula and certain areas in the south of the Ebro valley, while with the Calculated Map these areas are mainly found in the south of the Iberian System and the south of the Ebro valley.

The most significant change occurs in the cells between 101 and 200 Bq/m^3^, where it drops from 59% in the case of the P90 Potential Map to 35% in the Calculated Map. The reduction is this percentage is accompanied by a large group of the cells located in this category switching, on the Calculated Map, to the range between 301–400 Bq/m^3^ and cells of less than 100 Bq/m^3^.

Regarding the range of cells with concentrations below 100 Bq/m^3^, an increase in the number of cells is observed, going from 2% on the P90 Potential Map to 7% on the Calculated Map. This increase in cells is due to a large number of the cells identified in the CSN map as in the range between 101–200 Bq/m^3^ having moved to this range of lower concentrations. The area in this category lies mainly in the south of Spain in the Guadalquivir Valley and the Levante.

### 3.3. Assessment of the Degree of Identification of the Maps

To quantify the degree of identification of the Radon Potential Calculated Map with respect to radon concentrations, the cells are analyzed, identifying for each variable the percentage of failures or successes in each of the ranges. Table 4 reflects the degree of identification of each of the study variables.

#### 3.3.1. Degree of Identification Regarding Radon Concentrations

Regarding radon concentrations, both maps show a high capacity to identify cells with concentrations greater than 400 Bq/m^3^, but the Calculated Potential Map improves the data obtained with respect to the P90 Potential Map: it returns 68% correct identification of these areas compared to 64%.

The increase in the identification capacity of the Calculated Potential Map in areas with concentrations between 301–400 Bq/m^3^ is of special interest, from 20% to 3% reported by potential radon map CSN, Directive 2013/59/Euratom sets the first value as a reference level to be considered when devising National Action Plans against radon gas in order to define Radon Priority Areas that, with the proposed method, becomes easier to define.

In the intermediate concentration ranges, the identification capacity of the P90 Potential Map is superior to that of the Calculated Potential Map: In the range 201–300 Bq/m^3^ and 101–200 Bq/m^3^ it identifies appropriately 30% and 37% of the cells. The Calculated Potential Map correctly identifies 15% of cases.

The greater capacity of identification of the P90 Potential Map in these ranges of mean concentrations is mainly due to the scarcity of measurements made in these areas. As previously shown, a higher sampling density per cell more precisely defines the concentrations, and many of the cells identified as having average concentrations would move to another range of concentrations as the number of measurements in them increases. This greater identification capacity is also due to the fact that the CSN map assigned a high weight to the gamma radiation variable, while when creating the Calculated Potential Map, the weight of the variables was homogenized.

In the lower concentration ranges, the identification capacity of the Calculated Potential Map improves to 12% as against 3% of the P90 Potential Map.

#### 3.3.2. Degree of Identification Regarding the Rates of Exposure to Terrestrial Gamma Radiation

Regarding the rates of exposure to terrestrial gamma radiation, the P90 Potential Map has a greater capacity of identification (57%) as compared to the Calculated Potential Map (32%). However, when analyzing the different ranges in detail, it is found that this reduction in global accuracy is mainly due to low identification in the cells corresponding to the average rates (between 45 nGy/h and 122 nGy/h), because the data on radon concentration in this grid is limited. On the other hand, the increase in the identification of cells with higher rates is notable: in cells with more than 167 nGy/h the level of identification increases to 90%, and in cells between 123 nGy/h and 167 nGy/h it rises to 8%.

#### 3.3.3. Degree of Identification Regarding the Lithostratigraphies

With respect to lithostratigraphies, both maps correctly identify 47% of the cells. The differences emerge when analyzing the different classes associated with concentrations.

In the cells corresponding to lithostratigraphies associated with concentrations of more than 400 Bq/m^3^ (Class 9), the Calculated Potential Map increases identification to 96% as against 89% of the P90 Potential Map.

It is of particular interest that the Calculated Potential Map correctly identifies 36% of the cells associated with Classes 7 and 8 (lithostratigraphies linked to radon concentrations between 301 Bq/m^3^ and 400 Bq/m^3^), since the CSN map does not have the capacity to identify these areas. Again, Directive 2013/59/Euratom sets the value 300 Bq/m^3^ as the reference level for producing National Action Plans against radon gas.

The identification of cells associated with concentrations between 201 Bq/m^3^ and 300 Bq/m^3^ (Classes 4, 5 and 6) is similar in both maps: 27% in the case of the Calculated Potential Map and 31% of the P90 Potential Map.

The identification capacity of the Calculated Potential Map drops to 65% compared to 91% of the P90 Potential Map in Class 2 and 3 cells (lithostratigraphies associated with concentrations between 101 Bq/m^3^ and 200 Bq/m^3^). This is due to the existence of lithostratigraphies that were previously identified with intermediate concentrations, but now have come to be placed in the category of low concentrations: in Class 1 (lithostratigraphies associated with radon concentrations of less than 100 Bq/m^3^) the Calculated Potential Map has an accuracy of 80% as opposed to the null capacity of the P90 Potential Map.

## 4. Conclusions

In conclusion, it has been shown that:

- The Calculated Potential Map correctly identifies 12% of the cells in terms of the probability of finding a radon concentration in a given area, improving the percentage of the P90 Potential Map (which correctly identifies 10% of the cells).
- Regarding the probability of finding an exposure rate to terrestrial gamma radiation associated with a radon concentration, the P90 Potential Map properly identifies 57% of the cells, while the Calculated Potential Map identifies 32% of the cells. This is because when making the map, the CSN gave great weight to this variable, whereas when making the Calculated Potential Map, the weight of the study variables was homogenized.
- Regarding the probability of finding lithostratigraphies related to the greater or lesser presence of radon, both maps correctly identify 47% of the cells. In general, it is seen that the Calculated Radon Potential Map improves the identification of cells in terms of the probability of finding a radon concentration associated with a type of lithostratigraphy, since it homogenizes the ability to place a type correctly in all concentration ranges. Its identification capacity is markedly better in the ranges of higher concentrations (>300 Bq/m^3^) and lower concentrations (<100 Bq/m^3^).
- The Calculated Radon Potential Map in this study prepared from joining together the correlation maps shows that in 36% of the country there is a probability of finding radon concentrations higher than 300 Bq/m^3^ (17% above 400 Bq/m^3^ and 19% between 301 Bq/m^3^ and 400 Bq/m^3^). With this map, the areas of Spain with probable high radon concentrations (more than 300 Bq/m^3^) are precisely defined.

The map also identifies the areas with a probability of finding radon concentrations of between 100 Bq/m^3^ and 300 Bq/m^3^ more reliably, by homogenizing the weights of the variables. This range of concentrations is also of particular interest, as the WHO designates 100 Bq/m^3^ as the recommended reference level to start action plans against radon gas.

## Figures and Tables

**Table 1:** Equivalences and data grouping by ranges of values.

^222^Rn Concentration (Bq/m^3^)	Calculated Potential Radon Map (Value)	Pearson’s Correlation Coefficient (R)	Lithostratigraphies and Terrestrial Gamma Radiation Rates Intersections Value (Value)
>400	18 to 14	>+0.75	9
301–400	13 to 10	+0.74 to +0.26	7 to 8
201–300	9 to 7	+0.25 to −0.25	6 to 4
101–200	6 to 4	−0.26 to −0.74	3 to 2
<100	3 to 1	<−0.75	1

**Table 2:** Statistics on radon concentration data for Spain.

	Number of Measurements	Arithmetic Mean (Bq/m^3^)	Arithmetic Standard Deviation	Geometric Mean (Bq/m^3^)	Geometric Standard Deviation	1-st Quartile (Bq/m^3^)	Median (Bq/m^3^)	3-rd Quartile (Bq/m^3^)	Range (Bq/m^3^)	Skewness	Kurtosis
Spain	11,500	101	260.6	58	2.6	30	56	110	10–15,400	31.5	1497

**Table 3:** Mean concentration of ^222^Rn and number of measurements per 10 × 10 cell.

^222^Rn Concentration Arithmetic Mean (A.M) (Bq/m^3^)	No of 10 km × 10 km Cells (%)	No of Measurements per Cell (Average)	1 Measurements (%)	2 to 6 Measurements (%)	>6 Measurements (%)
>400	1	3.9	32	52	16
301–400	1	4.2	34	45	21
201–300	4	5.2	37	40	23
101–200	18	4.9	37	42	21
<100	76	2.8	50	41	9

**Table 4:** Degree of identification between the variables of the maps Potential Radon Map Calculated and P90 CSN.

**Radon Potential Calculated Map**								
**^222^** **Rn Concentration A.M** **(Bq/m^3^)**	**Success (%)**	**Failure (%)**	**Gamma Radiation Rate** **(nGy/h)**	**Success (%)**	**Failure (%)**	**Lithostratigraphies 1:200,000** **(Class)**	**Success (%)**	**Failure (%)**
>400	68	32	>167	90	10	9	96	4
301–400	20	80	123–167	8	92	8, 7	36	64
201–300	15	85	89–122	3	97	6, 5, 4	27	73
101–200	15	85	45–88	41	59	3, 2	65	35
<100	11	89	<48	0	100	1	80	20
	12	86		32	68		47	53
**Radon Potential Map P90 CSN**								
**^222^** **Rn Concentration A.M** **(Bq/m^3^)**	**Success (%)**	**Failure (%)**	**Gamma Radiation Rate** **(nGy/h)**	**Success (%)**	**Failure (%)**	**Lithostratigraphies 1:200,000** **(Class)**	**Success (%)**	**Failure (%)**
>400	64	36	>167	53	47	9	89	11
301–400	3	97	123–167	2	98	8, 7	0	100
201–300	30	70	89–122	31	69	6, 5, 4	31	69
101–200	37	63	45–88	72	28	3, 2	91	9
<100	2	98	<48	0	100	1	0	100
	10	90		57	38		47	53

## Data Availability

The data presented in this study are available in article.
